# Detailed Multiplex Analysis of SARS-CoV-2 Specific Antibodies in COVID-19 Disease

**DOI:** 10.3389/fimmu.2021.695230

**Published:** 2021-06-10

**Authors:** Siggeir F. Brynjolfsson, Hildur Sigurgrimsdottir, Elin D. Einarsdottir, Gudrun A. Bjornsdottir, Brynja Armannsdottir, Gudrun E. Baldvinsdottir, Agnar Bjarnason, Olafur Gudlaugsson, Sveinn Gudmundsson, Sigurveig T. Sigurdardottir, Arthur Love, Karl G. Kristinsson, Bjorn R. Ludviksson

**Affiliations:** ^1^Department of Immunology, Landspitali – The National University Hospital of Iceland, Reykjavik, Iceland; ^2^Faculty of Medicine, University of Iceland, Reykjavik, Iceland; ^3^Department of Clinical Microbiology, Landspitali – The National University Hospital of Iceland, Reykjavik, Iceland; ^4^Department of Infectious Diseases, Landspitali – The National University Hospital of Iceland, Reykjavik, Iceland; ^5^The Icelandic Blood Bank, Landspitali – The National University Hospital of Iceland, Reykjavik, Iceland

**Keywords:** COVID-19, SARS-CoV-2, antibodies, multiplex, IgG, IgA, IgM

## Abstract

A detailed understanding of the antibody response against SARS-CoV-2 is of high importance, especially with the emergence of novel vaccines. A multiplex-based assay, analyzing IgG, IgM, and IgA antibodies against the receptor binding domain (RBD), spike 1 (S1), and nucleocapsid proteins of the SARS-CoV-2 virus was set up. The multiplex-based analysis was calibrated against the Elecsys^®^ Anti-SARS-CoV-2 assay on a Roche Cobas^®^ instrument, using positive and negative samples. The calibration of the multiplex based assay yielded a sensitivity of 100% and a specificity of 97.7%. SARS-CoV-2 specific antibody levels were analyzed by multiplex in 251 samples from 221 patients. A significant increase in all antibody types (IgM, IgG, and IgA) against RBD was observed between the first and the third weeks of disease. Additionally, the S1 IgG antibody response increased significantly between weeks 1, 2, and 3 of disease. Class switching appeared to occur earlier for IgA than for IgG. Patients requiring hospital admission and intensive care had higher levels of SARS-CoV-2 specific IgA levels than outpatients. These findings describe the initial antibody response during the first weeks of disease and demonstrate the importance of analyzing different antibody isotypes against multiple antigens and include IgA when examining the immunological response to COVID-19.

## Highlights

- A multiplex based assay is as sensitive as the Elecsys^®^ Anti-SARS-CoV-2 assay in detecting SARS-CoV-2 specific IgG antibodies against N protein.
- SARS-CoV-2 specific antibodies class switch earlier to IgA than IgG in COVID-19 disease.
- Patients with high levels of SARS-CoV-2 specific IgA antibodies are more likely to be inpatients or ICU patients.

## Introduction

The need for a robust and clinically validated method to evaluate the serological immune response against the SARS-CoV-2 virus is of high importance. Both to elucidate the pathogenesis of COVID-19 and to evaluate the efficacy of vaccines. In Iceland, extensive testing, tracking, and surveillance for COVID-19 has been performed. However, the true prevalence of COVID-19 might be higher than the number of cases confirmed by RT-PCR, based on results from antibody screening of the Icelandic population, where 56% of individuals positive for SARS-CoV-2 antibodies had been diagnosed by RT-PCR (1).

The immunological memory formed after COVID-19 infection and the persistence of antibodies is of great interest. Studies have focused on antibodies against the spike protein, especially the receptor binding domain (RBD) situated at the spike1 (S1) subunit of the spike protein. Which is part of the virus that connects to the Angiotensin-converting enzyme 2 on the membrane of host cells (2). Studies have shown rapid generation of anti-RBD IgG (3), anti-S (4) and antibodies against the Nucleocapsid (N) protein (5). It has however been shown that antibodies against different epitopes of the SARS-CoV-2 virus (N protein and RBD) do not always correlate, indicating that it is not always sufficient to measure only one epitope (6). Cross-reacting antibodies that have been formed against other coronaviruses, such as SARS-CoV-1, MERS or other seasonal coronaviruses, can also be measured in tests specific for SARS-CoV-2 due to the homology of the proteins (7).

The aim of the study was to calibrate a multiplex assay examining the IgM, IgG, and IgA antibody levels against the immunogenic antigens, i.e. the N, RBD and S1 proteins, of SARS-CoV-2 using the commercial Elecsys^®^ Anti-SARS-CoV-2 assay from Roche. The calibrated assay was then used to analyze patients´ antibody profiles and correlate them with disease outcomes.

## Materials and Methods

### Conjugation of Microspheres

RBD, N protein, and S1 (Trenzyme, Germany) were conjugated to MagPlex^®^ Microspheres (Luminex, USA) at a ratio of 3µg protein/1*10^6^ microspheres with NHS (50mg/ml) and EDC (40mg/ml). Patient serum samples and serum pool were incubated with microspheres at a 1:100 dilution, subsequently with secondary antibodies for IgM, IgG and IgA and analyzed on a Bioplex-200 machine (Bio-rad, USA). A more detailed protocol can be found in the supplement.

### Patient Samples

Clinical serum samples for calibration of the multiplex assay were prospectively collected from the Department of Clinical Microbiology. One hundred samples (disease not confirmed with RT-PCR) were collected, 50 positive and 50 negative as measured by Elecsys^®^ Anti-SARS-CoV-2 on the Cobas e 411 analyzer (Roche diagnostics).

Negative controls, used to test for cross reactivity, were 36 pre-pandemic serum samples collected in 2019 by the Department of Immunology.

The multiplex assay was normalized using a serum pool (MFI of sample/MFI of serum pool), consisting of serum from 46 healthy blood donors, collected in august 2018, aliquoted and stored at -80°C.

All COVID-19 patient samples received by the Department of Immunology (251 samples, from 221 RT-PCR confirmed COVID-19 patients), from February – May 2020 were analyzed. Patient demographics and sampling times from self-reported start of symptoms are shown in **Supplementary Table 1**.

All COVID-19 patients received medical care and follow-up for the duration of their illness and/or isolation.

The study was approved by The National Bioethics Committee (VSN-20-169) and the Data Protection Authority.

### Statistical Analysis

Results are presented as mean ± standard deviation (SD). Two-way ANOVA and Kruskal-Wallis tests were used when appropriate. Differences were considered statistically significant when p<0.05. Analysis was performed on GraphPad Prism 9 (GraphPad, USA).

## Results and Discussion

### The Multiplex Serologic Assay Is of High Reliability and Validity

**Figure 1:**
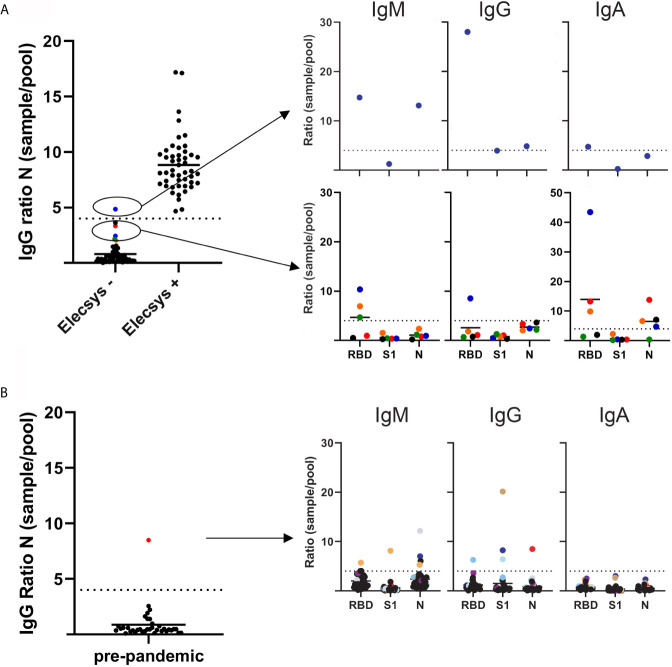
SARS-CoV-2 antibody levels as measured by a multiplex based assay and calibrated against the Elecsys^®^ Anti-SARS-CoV-2 assay (Roche diagnostics). **(A)** 100 serum samples previously analyzed by Elecsys^®^ assay (depicted on the x axis as negative and positive), were received from the Department of Clinical Microbiology, and SARS-CoV-2 IgG antibodies against the N protein were analyzed blindly by multiplex (left panel). Right top panel depicts the single sample that had a SARS-CoV-2 IgG >4 sample/pool ratio for the N protein. Bottom right panel depicts the samples that showed a >2 but <4 than 4 sample/pool ratio by multiplex. **(B)** Left panel depicts 36 serum samples received in 2019 by the Department of Immunology. SARS-CoV-2 IgG antibodies against the N protein were analyzed by multiplex. Right panel depicts the serum/pool ratio of the serum samples against RBD, S1 and N proteins. Samples above the cut-off level are color coded.

SARS-CoV-2 IgG antibodies against N protein were blindly analyzed using the multiplex assay in 100 prospectively collected samples from the Department of Clinical Microbiology, previously analyzed by the Elecsys^®^ Anti-SARS-CoV-2 assay (**Figure 1A**).

The samples were normalized by dividing the median fluorescence intensity (MFI) of the samples with the MFI from a serum pool provided by the Icelandic Blood bank. After normalizing measurements from the multiplex assay, a sample/pool ratio of 4 for IgG antibody ratio against the N protein was found to discriminate between IgG positive and negative samples, with 99 of 100 results in agreement with the Elecsys^®^ Anti-SARS-CoV-2 assay (**Figure 1A**). Therefore, a ratio of 4 was used as a cut-off value to discriminate between positive and negative samples. This cut-off value was applied to the S1, RBD proteins and against the IgA and IgM isotypes as well.

The single discordant sample that was negative in the Elecsys^®^ but positive in the multiplex had a sample/pool ratio >4 (4.83) in IgG for N protein. The multiplex also showed a >4 IgM ratio against both RBD and N protein (14.74 and 13.12, respectively) and a >4 IgG and IgA RBD ratio (28 and 4.74, respectively) (**Figure 1A**). These results indicate that this sample came from an individual early in disease that is undergoing class switching. Additionally, differences in incubation times between Elecsys and the multiplex assay (9 min vs. 30 min) may play a role, with a longer incubation time allowing the detection of antibodies with a lower affinity.

Several negative samples were close to the cut-off (sample/pool ratio 2-4 in multiplex assay). A ratio >4 for IgM RBD and IgA RBD was detected in 3 out of the 4 samples and 1 out of 4 samples had >4 ratio of IgG RBD. These results suggest that these samples were undergoing class switching from IgM antibodies towards IgG and IgA, suggesting that a ratio of 4 might be too stringent, resulting in false negative outcomes (**Figure 1A**).

SARS-CoV-2 IgG specific antibodies against N protein were analyzed in 36 pre-pandemic serum samples. One case had a sample/pool ratio >4 (**Figure 1B**). Further analysis revealed a different pattern than for the COVID-19 samples, with no increase in IgM or IgA levels. This might reflect a previous infection with a different coronavirus due to the known potential cross-reactivity of the N protein for this virus (8).

Using results from the Elecsys^®^ assay and pre-pandemic samples as true positive and negative, the calculated sensitivity and specificity of the multiplex assay for IgG against N protein by applying a >4 sample/pool ratio, was 100% and 97.7%, respectively.

These results indicate that the multiplex assay is a sensitive and specific method for detecting SARS-CoV-2 specific IgG antibodies against the N protein.

### Class Switching in SARS-CoV-2 Infected Individuals Occurs Faster for IgA Than for IgG

**Figure 2:**
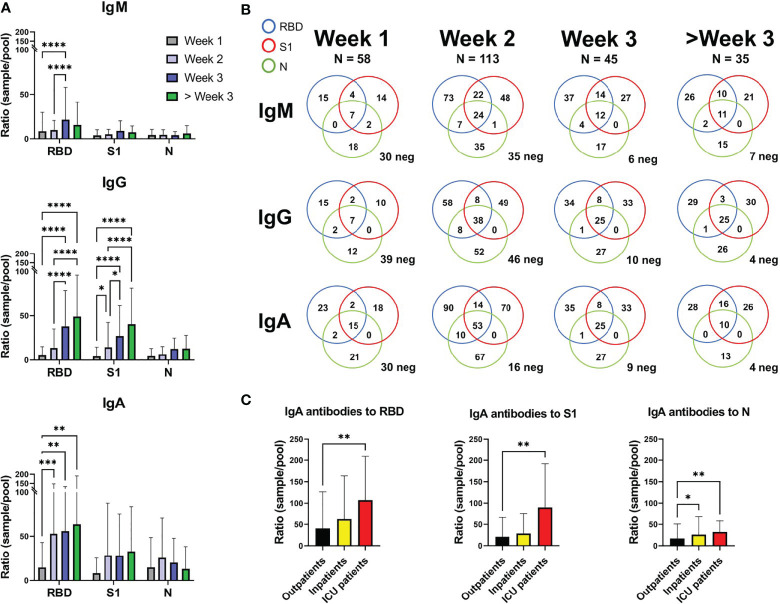
SARS-CoV-2 specific antibody levels against the RBD, S1, and N proteins in 221 COVID-19 patients. **(A)** Depicting the mean and standard deviation in sample/pool ratio of SARS-CoV-2 antibody levels for IgM, IgG, and IgA for weeks 1, 2, 3 and over 3 weeks. **(B)** Venn diagram showing the number of patients with a ratio >4 for IgM, IgG, and IgA against the RBD, S1 and N proteins. Patients below the cut-off value of 4 in ratio against all proteins are depicted as neg in the bottom right corner of each diagram. 35 out of the 251 samples were below 4 for all the proteins, for the three antibody isotypes. **(C)** The IgA sample/pool ratio in COVID-19 outpatients (n=168), inpatients (n=40), and ICU patients (n=13). *, **, *** and **** correspond to p=<0.05, p=<0.01, p<0.001 and p=0.0001, respectively.

SARS-CoV-2 specific IgM, IgG, and IgA antibodies against RBD, N, and S1 were analyzed in 251 samples from 221 COVID-19 patients using the multiplex assay (**Figure 2A**). For RBD, a significant rise in antibody levels for IgM, IgG, and IgA was seen when comparing samples taken during week 1, 2, and week 3. For IgA, samples from week 1 showed a significantly lower ratio than weeks 2 and 3. For S1, the ratio increased significantly for IgG between weeks (**Figure 2A**).

In samples from the first week of disease, 30 of 58 samples were considered IgM negative (ratio below 4) for all three proteins (**Figure 2B**). For IgG and IgA, 39/58 and 30/58 were negative, respectively. However, during week 3, only 6 of 45 samples were IgM negative for all three proteins, while 10 and 9 were negative for IgG and IgA, respectively. During the third week the majority of patients had thus undergone class switching, which seemed to occur faster for IgA than IgG (16 negative for IgA and 46 negative for IgG during week 2).

Out of 221 COVID-19 patients, 35 (15.8%) cases did not reach the defined cut-off level for IgM, IgG, or IgA for any of the three proteins (17/58 week 1, 12/113 week 2, 6/45 week 3, 0/35 > week 3). However, a cut-off ratio of >4 ratio might be unnecessarily restrictive, decreasing sensitivity early on in disease.

Patients admitted to intensive care showed higher IgA ratios for RBD and S1 when compared to the outpatient group, while both inpatients and intensive care patients showed higher IgA ratios for N protein than outpatients (**Figure 2C**). IgG and IgM did not show any statistical differences between patient groups. Most of the intensive care patients were admitted within the first 2 weeks of disease (1 during week 1, 8 during week 2, 4 during week 3 with an average of 12.69 days after self-reported start of symptoms, **Supplementary Table 1**).

Our findings are in agreement with previous reports on a high IgA levels, both total (9) and SARS-CoV-2 specific IgA (5, 10, 11), in patients with more severe COVID-19 disease. High viral load in the respiratory mucosa has been associated with more severe symptoms (12) and a more robust IgA response might also indicate a more aggressive disease and worse outcome.

There was a clear difference in the antibody profile between samples from patients infected with SARS-CoV-2 and samples from patients with cross-reactive antibodies against one or more of the proteins. Showing that this method can discriminate between individuals that are infected with SARS-CoV-2, and non-infected individuals showing cross-reactive antibodies.

This study examines multiple antibody isotypes, against multiple SARS-CoV-2 antigens in 221 COVID-19 patients with varying disease severity at early timepoints in the disease were examined. Of the 3 antigens tested, RBD seemed to be the most immunogenic. Interestingly, following the initial IgM response, IgA class switching occurred at a more rapid rate than IgG. Finally, a more robust IgA response was found in patients with severe disease requiring ICU admission.

## Conjugation of microspheres

SARS-CoV-2 proteins S1, RBD, and N (all from Trenzyme, Konstanz, Germany), were conjugated to MagPlex^®^ Microspheres (Luminex Corporation, Austin, Texas, USA) at a ratio of 3µg protein/1*10^6^ microspheres. Microspheres were washed using a magnet (vortex and sonication was included with each wash) twice with an activation buffer (100mM NaH_2_PO_4_ in water, pH 6.0). Microspheres were resuspended in activation buffer, Sulfo-NHS (50mg/ml) and EDC (40mg/ml) were added to the microspheres and incubated in dark on a rotator for 20 min. Microspheres were washed three times with coupling buffer (50mM MES, pH 5.0). Microspheres were resuspended in coupling buffer, protein added and incubated on rotor for two hours. Microspheres were washed three times and resuspended with blocking buffer (PBS, 1% newborn bovine serum (NBBS), 0.08% Azide, sterile), and stored in dark and 4°C until use.

## Assay

Briefly, 2500 microspheres for each protein were added to each well in 3x96 well plates (one for each isotype) and washed in a Bio-Plex Pro (Bio-Rad, Hercules, California, USA) wash station. Serum samples, both the serum pool and patient samples, were diluted 1/100 with PBS with 2% NBBS and incubated with the microspheres for 30 min/RT/shaking in the dark. Plates are washed and secondary antibodies are added in a 1/100 dilution (diluted in PBS-T with 2% NBBS), then incubated for 30 min/RT/shaking in the dark (anti-IgA-PE is from Southern Biotech, Birmingham, Alabama, anti-IgG-PE and anti-IgM-PE from Jackson ImmunoResearch, West Grove, Pennsylvania, USA). Microspheres are resuspended after wash in PBS-T with 2% NBBS and analyzed on a Bio-plex 200 (Bio-Rad).

## Data Availability Statement

The raw data supporting the conclusions of this article will be made available by the authors, without undue reservation.

## Ethics Statement

The study was approved by The National Bioethics Committee (VSN-20-169) and the Data Protection Authority. Written informed consent for participation was not required for this study in accordance with the national legislation and the institutional requirements.

## Author Contributions

SB and HS: designed the research, measured samples, analyzed data, and wrote the manuscript. SS and BL: designed research and wrote the manuscript. EE: measured samples. GBj: designed research and wrote the manuscript. BA, GBa, AL, and KK: provided serum samples and Elecsys measurements. AB and OG: provided clinical data. SG: provided the serum pool. All authors contributed to the article and approved the submitted version.

## Funding

This work was supported in part by The Student Innovation Fund. The funding body had no role in the design of the study, collection, analysis, interpretation of data or writing of the manuscript.

## Conflict of Interest

The authors declare that the research was conducted in the absence of any commercial or financial relationships that could be construed as a potential conflict of interest.
