# Erratum: Detailed Multiplex Analysis of SARS-CoV-2 Specific Antibodies in COVID-19 Disease

**DOI:** 10.3389/fimmu.2021.733897

**Published:** 2021-07-12

**Authors:** Frontiers Production Office

**Affiliations:** Frontiers Media SA, Lausanne, Switzerland

**Keywords:** COVID-19, SARS-CoV-2, antibodies, multiplex, IgG, IgA, IgM

Due to a production error, there was a mistake in **Figure 1** as published. The image was published in grayscale instead of color. The corrected **Figure 1** appears below.

**Figure:**
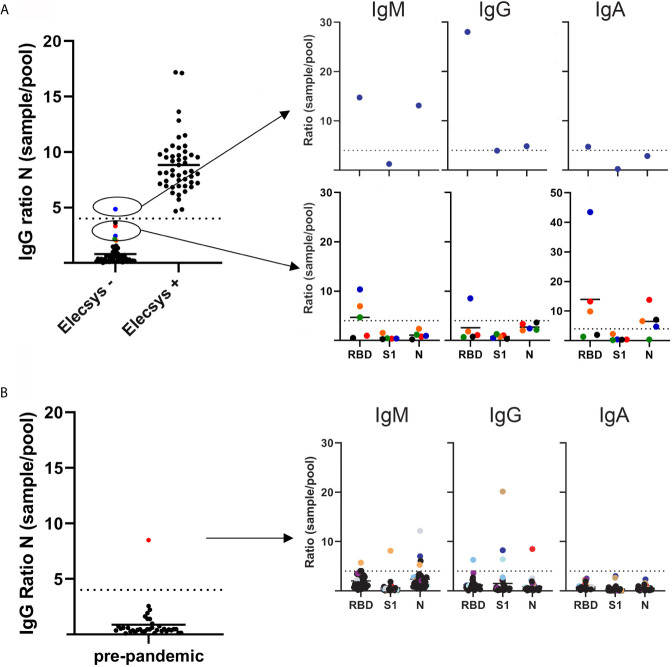
SARS-CoV–2 antibody levels as measured by a multiplex based assay and calibrated against the Elecsys® Anti-SARS-CoV-2 assay (Roche diagnostics). **(A)** 100 serum samples previously analyzed by Elecsys® assay (depicted on the x axis as negative and positive), were received from the Department of Clinical Microbiology, and SARS–CoV–2 IgG antibodies against the N protein were analyzed blindly by multiplex (left panel). Right top panel depicts the single sample that had a SARS–CoV–2 IgG >4 sample/pool ratio for the N protein. Bottom right panel depicts the samples that showed a >2 but <4 than 4 sample/pool ratio by multiplex. **(B)** Left panel depicts 36 serum samples received in 2019 by the Department of Immunology. SARS-CoV-2 IgG antibodies against the N protein were analyzed by multiplex. Right panel depicts the serum/pool ratio of the serum samples against RBD, S1 and N proteins. Samples above the cut-off level are color coded.

The publisher apologizes for this mistake. The original version of this article has been updated.

