# Supplementary material for: Detailed Multiplex Analysis of SARS-CoV-2 Specific Antibodies in COVID-19 Disease
Source: Front Immunol. 2021 Jun 10;12:695230. doi: 10.3389/fimmu.2021.695230 (PMC8222737; doi:10.3389/fimmu.2021.695230)
Supplement: Supplementary file 1 [file DataSheet_1.docx]

**Conjugation of microspheres**

SARS-CoV-2 proteins S1, RBD, and N (all from Trenzyme, Konstanz, Germany), were conjugated to MagPlex^®^ Microspheres (Luminex Corporation, Austin, Texas, USA) at a ratio of 3µg protein / 1*10^6^ microspheres. Microspheres were washed using a magnet (vortex and sonication was included with each wash) twice with an activation buffer (100mM NaH_2_PO_4_ in water, pH 6.0). Microspheres were resuspended in activation buffer, Sulfo-NHS (50mg/ml) and EDC (40mg/ml) were added to the microspheres and incubated in dark on a rotator for 20 min. Microspheres were washed three times with coupling buffer (50mM MES, pH 5.0). Microspheres were resuspended in coupling buffer, protein added and incubated on rotor for two hours. Microspheres were washed three times and resuspended with blocking buffer (PBS, 1% newborn bovine serum (NBBS), 0.08% Azide, sterile), and stored in dark and 4°C until use.

**Assay**

Briefly, 2500 microspheres for each protein were added to each well in 3x96 well plates (one for each isotype) and washed in a Bio-Plex Pro (Bio-Rad, Hercules, California, USA) wash station. Serum samples, both the serum pool and patient samples, were diluted 1/100 with PBS with 2% NBBS and incubated with the microspheres for 30 min/RT/shaking in the dark. Plates are washed and secondary antibodies are added in a 1/100 dilution (diluted in PBS-T with 2% NBBS), then incubated for 30 min/RT/shaking in the dark (anti-IgA-PE is from Southern Biotech, Birmingham, Alabama, anti-IgG-PE and anti-IgM-PE from Jackson ImmunoResearch, West Grove, Pennsylvania, USA). Microspheres are resuspended after wash in PBS-T with 2% NBBS and analyzed on a Bio-plex 200 (Bio-Rad).

| Patient demographics |  |  |
| --- | --- | --- |
| N=221 |  | Average days after self reported start of symptoms |
|  |  |  |
| Age | 52.71 (37.69 - 67.72) |  |
| Female | 130 |  |
| Male | 91 |  |
|  |  |  |
| Outpatients | 168 | 15.88 (4.25 - 27.51 |
| Inpatients | 40 | 9.63 (4.86 - 14.40) |
| ICU patients | 13 | 12.69 (8.79 - 16.59) |
|  |  |  |
| Distribution of sampling time |  |  |
| Weeks from self reported start of symptoms | n |  |
|  |  |  |
| Week 1 (day 0-7) | 58 | 5.53 (4.08 - 6.99) |
| Week 2 (day 8-14) | 113 | 10.88 (8.79 - 12.96) |
| Week 3 (day 15-21) | 45 | 17.22 (15.19 - 19.25) |
| Week >3 (day >21) | 35 | 33.20 (19.07 - 47.33) |

**Supplementary table 1.** Patient demographics showing the mean age in years (±SD), number of females, males, outpatients, inpatients, intensive care (ICU) patients and average days (±SD) after self reported start of symptoms of each individual patient group.
